# Performance indicators for radiation protection management: suggestions from the European Society of Radiology

**DOI:** 10.1186/s13244-020-00923-1

**Published:** 2020-12-09

**Authors:** European Society of Radiology (ESR), Peter Mildenberger, Adrian P. Brady, Mehmet Onur, Graciano Paulo, Daniel Pinto Dos Santos, David Howlett, Guy Frija

**Affiliations:** European Society of Radiology (ESR), Am Gestade 1, 1010 Vienna, Austria

## Abstract

In 2013, the new European Basic Safety Standards Directive 2013/59/Euratom (BSS Directive), which defines the new legal framework for the use of ionising radiation in medical imaging and radiotherapy, was published. In 2014, the ESR EuroSafe Imaging Initiative was founded with a goal in mind “*to support and strengthen medical radiation protection across Europe following a holistic, inclusive approach”.* To support radiology departments in developing a programme of clinical audit, the ESR developed a Guide to Clinical Audit and an accompanying audit tool in 2017, with an expanded second edition released in 2019 and published under the name of Esperanto – ESR Guide to Clinical Audit in Radiology and the ESR Clinical Audit Tool, 2019. Audits represent specific aspects at a certain point in time, usually with retrospective evaluation of data. Key performance indicators (KPIs), on the other hand, are intended to enable continuous monitoring of relevant parameters, for example to provide warnings or a dashboard. KPIs, which can, for example, be recorded automatically and visualised in dashboards, are suitable for this purpose. This paper will discuss a selection of indicators covering different areas and include suggestions for their implementation.

## Key points

- The use of ionising radiation in medical imaging and radiotherapy underlies the European Basic Safety Standards Directive.
- In addition to clinical audit, key performance indicators enable continuous monitoring of relevant parameters.
- This paper discusses several indicators and suggests ways for their implementation.

## Introduction

Medical radiation exposure submits the population, as a whole, to a significant radiation dose approximately equivalent to natural radiation exposure. Therefore, efforts to reduce overall radiation exposure and avoid unnecessary or inadvertent exposure are important for public health. Efforts to control exposure are being intensively pursued by various organisations.

The so-called *Bonn Call for Action*, which was published at the IAEA and the WHO conference in 2012 in Bonn (Germany), is of central importance in this context. A total of ten objectives was defined [1]. Two of these targets specifically address the issue of radiation protection in association with diagnostic imaging studies:

These goals are:

- Goal 7: Improve prevention of medical radiation incidents and accidents
- Goal 8: Strengthen radiation safety culture in health care

In 2013, the new European Basic Safety Standards Directive 2013/59/Euratom (BSS Directive), which defines the legal framework for the use of diagnostic and interventional radiological procedures, was published [2, 3].

The European Society of Radiology (ESR) is the largest professional scientific society working with other international bodies and organisations, e.g. the European Commission and HERCA (Heads of the European Radiological Competent Authorities), on a European level to contribute to a high level of radiation protection across Europe and to minimise radiation exposure in diagnostic and interventional imaging procedures. In 2014, the ESR EuroSafe Imaging Initiative was founded with this goal in mind: “*to support and strengthen medical radiation protection across Europe following a holistic, inclusive approach”* [4].

EuroSafe Imaging pursues this mission with a variety of activities (in conjunction with other groups within the ESR), such as the development and dissemination of clinical decision support systems, improved information for patients, the development of a clinical audit guide and a tool, establishing a collection of European dose reference levels (DRLs). In 2018, the second version of the EuroSafe Imaging Call for Action was published. Action item five is the development of performance indicators in radiation protection management [5].

## Performance indicators

Assessing the performance of an organisation or of processes requires categorial or quantitative values. Such performance indicators are used in various areas to measure financial and non-financial aspects of activity. Key performance indicators (KPIs) are measures of how well various criteria are met and must be adapted to the respective question. The continuous evaluation of such measurements facilitates assessment of organisational development and the achievement of objectives. Typical areas for the use of KPIs include:

- Patient safety and quality of care
- Customer service
- Operations management
- Financial management

Individual KPIs can be grouped thematically, and compliance with KPIs in corresponding categories can then be indicated with so-called Balanced Scorecards [6–8].

The Boston MGH (Massachusetts General Hospital)/Harvard group has been active in the development of radiology-specific KPIs and describes numerous criteria covering a wide range of quality characteristics. These include various functions such as equipment usage, staff development, IT support, training and a number of other parameters. However, radiation protection is not the specific focus of their publication [8].

The application of such measuring indicators in the field of radiation protection should include various aspects, such as patient safety, personnel safety, image quality and clinical outcome. This requires an appropriate infrastructure that takes into account the different stakeholders, including radiologists and radiographers, medical physicists, manufacturers, patients and others. Results can be obtained for individual investigations, for long-term observation, and also for risk assessment. A quality/safety index can also be calculated on the basis of corresponding measurement figures. Typical questions in radiology are, for example, the range of frequency of need to repeat exposures (re-take range), whether a wrong patient has been examined, whether artefacts are recorded, or adverse effects of the administration of contrast agents or dose events are documented and analysed [9–12].

Due to their structure, and the time required to complete them, regular audits are not a tool for timely monitoring of process quality. Therefore, the selection of KPIs for continuous monitoring of radiation protection is important. Continuous recording of indicators can be used, for example, to supply a dashboard with values or to issue timely warnings.

When selecting such KPIs, a distinction must be made between radiation exposure of patients and staff. In addition, a distinction can be made with regard to measures that take place before, during or after an examination. A distinction should also be made in terms of the availability and applicability of indicators. Some indicators can be defined as standard, and others can be classified as advanced.

Continuous monitoring of performance indicators should be as free as possible from manual input. Criteria that are automatically collected from information systems as part of data collection are therefore preferable. For example, this could be the documentation of the justifying indication before the examination or the recording of dose values after the examination has been completed.

## Requirements for performance indicators

Different criteria can be used to assess the performance indicators. Several examples of such criteria are listed and explained below:

- *Automatisation* Automatic recording of values is advantageous compared to manual recording and less error-prone; an example of this is the continuous documentation of dose exposure by means of DICOM Radiation Dose SR objects, which can be registered, processed and evaluated automatically in a database.
- *Availability* The availability of the data to be selected should be representative in order to achieve meaningful results. Using the example of dose exposure, this means that the majority of devices should be able to transfer values to a departmental dose registry in an automated manner.
- *Consistency* It should be clear which data are collected and how they are documented in order to avoid differences in the way they are collected and interpreted.
- *Sufficiency of events for statistical analysis* In particular, for long-term assessment it is important to have sufficient data available to achieve statistically meaningful results.
- *Impact* Performance indicators should be focused on relevant clinical topics and should be relevant to both patient and staff safety and patient outcomes.
- *Reproducibility and stability* Indicators must be stable and reproducible
- *Usability* The relevant values should be easy to capture and should be unambiguous.

## Overview of performance indicators for radiation protection

Templates for performance indicators are available from many different institutions, including individual universities, professional societies, national institutions and the association of European supervisory authorities (HERCA) [13–17]. HERCA, for example, advocates to monitor the justification process, the qualification of staff, adherence of selected procedures to national and international guidelines or verification of similar imaging conducted recently [16].

### Compliance with appropriateness criteria

The justification for radiological examinations is a core topic of the BSS Directive and is correspondingly strongly represented within the ESR Clinical Audit Tool templates. The quality of the referral and appropriateness according to guidelines is important areas not as of yet addressed within legislation. It is known from various studies that in 20–25% of cases, even if an appropriate indication has been verified, the examination is not carried out in accordance with relevant guidelines [18, 19]. A monitoring of the appropriateness rate, at least for high-dose studies, could be used for auditing.

### Retake rate

The quality of individual examinations can be influenced by various factors, such as the patient, movement artefacts, exposure problems. The decision of whether to repeat the exposure is made by the radiographers and/or radiologists. The frequency of retakes can therefore be a relevant quality feature [20].

### Monitoring artefacts

Artefacts can significantly limit the informative value of radiological examinations, for example extracorporeal foreign bodies that overlap essential parts of the region to be examined or prostheses that impair the assessment of the lymph node stations in the pelvis. The detection of such artefacts and the impact in terms of limitation of significance or repetition rate may be a relevant quality indicator.

### KPIs for monitoring imaging equipment

The quality of the examination equipment is integral to the performance of radiology departments. Equipment hardware and software, for example dose-reducing reconstruction algorithms for computed tomography, and also the age of the devices themselves, are key issues. The ESR has developed recommendations also relating to the renewal of radiological equipment. Monitoring of departmental infrastructure with regard to the fulfilment of these criteria can be a relevant quality indicator [21].

### KPIs for monitoring protective tools

The availability and use of radiation protection clothing and equipment is one of the most important aspects in the optimisation of radiation protection. A departmental map with the number of protective devices (lead aprons, lead glass spectacles, thyroid protection, etc.), date of acquisition and characteristics, indicating the type and date of quality control procedures, the person responsible and the type of storage used, could be used for monitoring and auditing, combined with personal dosimetry recording.

### Indicators for personalised feedback

In addition to measures to improve radiation protection for the patient, optimisation of radiation protection also includes measures to improve radiation protection for staff. In addition to the use of radiation protection tools, individual experience and handling, especially in interventional radiology, are decisive. Individualised observation and documentation of workflows are therefore useful in order to promptly recognise any influences on the individual radiation exposure of equipment users. This can be done, for example, by real-time measuring systems, thus enabling personalised feedback [22].

### Indicators for patient feedback

Surveys regarding patients’ feedback about availability and clarity of radiation protection information in radiology departments may be important. Awareness in radiology departments of the patients’ knowledge, expectations and recommendations regarding radiation protection policies may be helpful in reducing the patients’ anxiety about radiation exposure and the management of radiation protection strategies in departments.

In the following list, various KPIs are proposed for such a task.

## Patient-centred KPIs

1. In advance of an exposure
  Justification of exposures (standard)—can be provided by RIS
  Compliance with Appropriateness Criteria, e.g. iGuide (advanced)
  Informing patients about the quantity of exposure and available alternate imaging methods
  Review of patients’ past examinations and dose history
2. During examination
  Documentation of retake-rate and their reasons (standard)
  Registration of accidental/unintended exposures (standard)
  Assessing specific patient factors (e.g. paediatric age, scoliosis, patients with high BMI, patients with trauma, etc.)
3. Post-exposure
  Local dose registry (standard)—provided by many RIS or specialised add-ons
  Benchmarking with regional or supra-regional registries (advanced)
  Timely and regular analysis of accidental/unintended exposures (standard)
  Registration of dose exposures in dose monitoring systems which result from interventions performed in departments other than radiology. (Awareness of radiation protection principles in radiology departments is usually good; however, procedures using ionising radiation are being increasingly performed in other departments (e.g. angiography in cardiology and fluoroscopic examinations in urology, gastroenterology, orthopaedics, etc.). Radiation protection processes may not be as well-developed in these departments. Integration of other departments with radiology in terms of dose registration will also be helpful for measuring cumulative dose and supporting decision-making about subsequent imaging examinations of patients in radiology departments.)

## Personnel-centred KPIs

- Online monitoring of eye lens doses (advanced)
- Staff dosimetry audit (standard) monitoring the impact of medical physics expertise, e.g. rate of protocols optimised (advanced).

A comprehensive overview on radiation protection-specific KPIs is shown in Table 1.

**Table 1:** The development of the age of equipment in one department over two years, OUT means, that the age of a specific device is out of range of the ESR requirements [21]

Modality	MR	CT	PET-CT	Angio	Mammo	RG	Fluoro	US	Bone dens
Number of modalities	6	4	1	2	2	6	1	5	1
Mean Age 2017	9	8,5	9	17,5	8,5	13,7	18	7	7
Mean Age 2019	11	10,5	11	5,5	10,5	13,2	20	9	9
OUT 2017	1	1	0	1	0	4	1	0	0
OUT 2019	1	2	0	1	1	3	1	4	0

## ESR clinical audit tool

As part of the BSS Directive, the regular performance of quality assessment within the framework of clinical audits is mandatory, "*according to national procedures*". In accordance with the legal framework, the performance of clinical audits has therefore been compulsory since 2018. These audits can help in the evolution of daily procedures. Audits may focus on topics related to radiation protection and the requirements of the BSS Directive or may be performed on a wider variety of clinical and technical topics. Audits can be carried out internally or as external audits, for example as part of a certification process [2, 23, 24].

Effective clinical audits can help in optimising patient care, experience and outcomes, with the results of the audits compared against standards. As part of the cycle of re-audits (the audit cycle), the progress of any potential quality improvement can then be assessed. To support radiology departments in developing a programme of clinical audit the ESR developed a Guide to Clinical Audit and accompanying audit tool in 2017, with an expanded second edition released in 2019 and published under the name of Esperanto – ESR Guide to Clinical Audit in Radiology and the ESR Clinical Audit Tool [25]. This is now available for general use on the ESR website. In total, 23 regulatory and 7 clinical audit templates are outlined in a step-by-step manner in this ESR tool, with further guidance provided on performing audits beyond these 30 [25, 26]. As part of this clinical audit tool, templates are provided, describing the necessary information and the steps to complete the audit. The majority of the topics in the ESR Clinical Audit Tool relate to radiation protection, with templates aligned to key areas as defined within the BSS Directive. In general, auditing specific targets requires measurements, which can be quantified or observations, which can be categorised. KPIs are particularly suitable and predestined for this.

In the field of radiation protection in particular, clinical audits usually need to be undertaken regularly and repeated to demonstrate continuing compliance with targets—as such the acceptance of the method and content is of particular importance. Audit should be *“Achievable, Local, Practical, Inexpensive, Non-threatening, and Easy (ALPINE)”* [25].

KPIs are intended to monitor specific aspects in a continuous form and activate alarms, if some values are outside a normal range. Therefore, the intention is different from audits, which represent a snapshot at a given time point, also including data from retrospective analysis.

During the ESR pilot study carried out in 2017, 5 “essential” topics out of a total of 17 were identified and subjected to trials across a network of EuroSafe Imaging Star departments [25, 26]:

- What is the mechanism for record keeping and retrospective analysis of adverse incidents?
- What is the departmental mechanism to confirm the non-pregnancy status of female patients?
- Is there a written protocol for who may be responsible for justification of CT studies?
- What mechanism is used to evaluate patient dose in high-dose procedures?
- How old is the equipment in your department?

The pilot study found that the chosen topics were relevant, the templates were straightforward and easy to use, and the process was time efficient [13].

## Experiences from use cases and ESR survey

### Use case: renewal of equipment

**Fig. 1:**
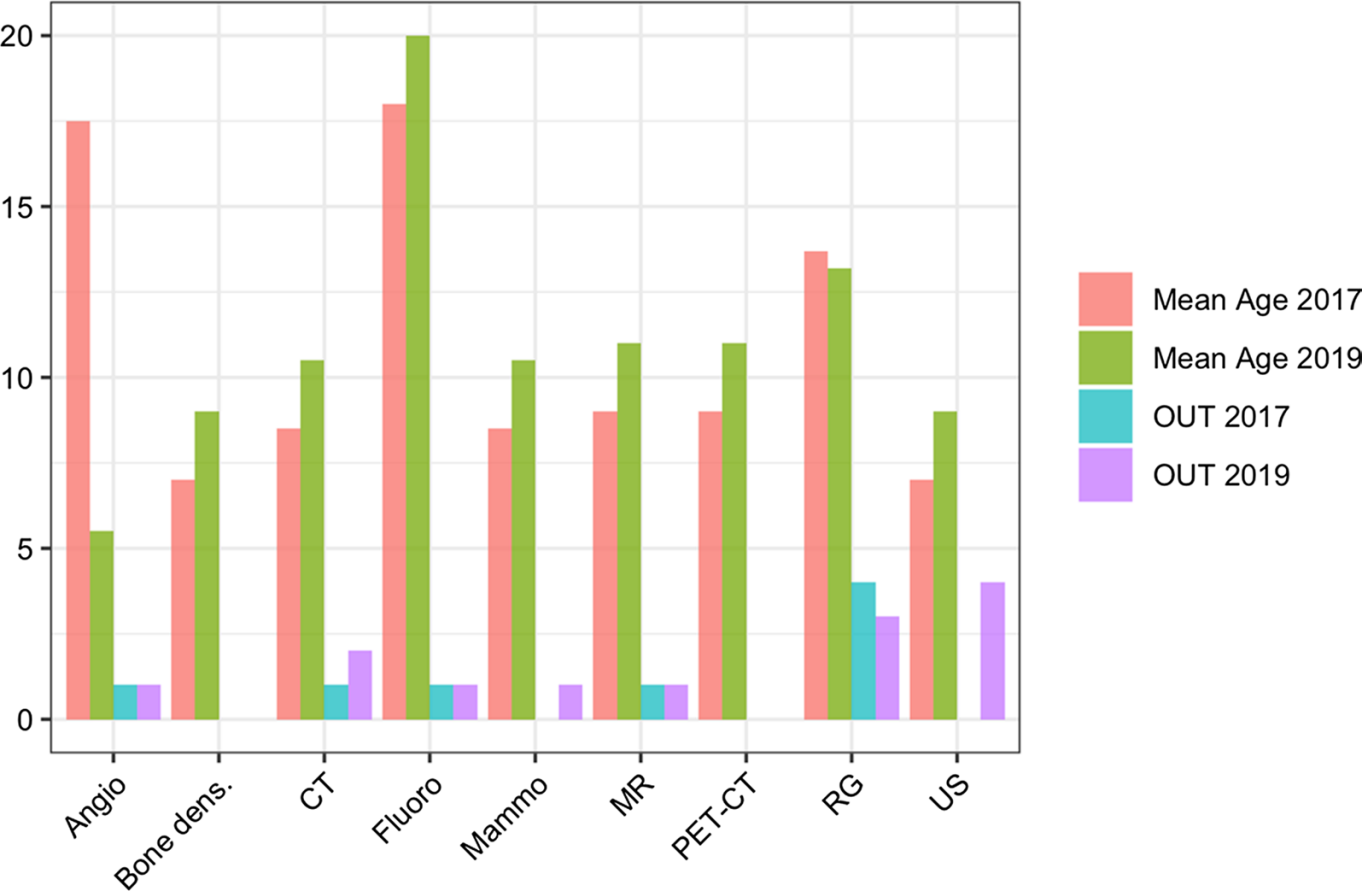
Increasing age of modalities in a department with the consequence that without appropriate investments a relevant part gets outside the ESR recommendations, e.g. USA [21]

ESR published a paper on renewal of radiology equipment in 2014 [21]. There are different criteria for when modalities should be renewed based on frequency of use and/or number of examinations per year. Using such criteria, it is possible to monitor the infrastructure in a department. In the given use case (Table 2, Fig. 1), it is obvious that there is limited reinvestment in the monitored time period from 2017 to 2019. In this period, just two modalities have been renewed (1 angiography, 1 radiography), which results in a fulfilment of ESR recommendations in 2017 at a level of 71% and a decline in fulfilment to 54% in 2019.

**Table 2:** Comprehensive overview on radiation protection-specific KPIs listing topics and indicators for measurements and validation

Workflow	Topic	Indicator	Definition
Order			
	Inappropriate orders CT MRI	Number of patients and %	If there is a CDS: data extraction If there is not an automatic data collection: retrospective review of 100 CT/most frequent indication (head, chest, abdomen, MSK) every year
	Inappropriate orders done CT MRI	Number of patients and %	If there is a CDS: data extraction If there is not an automatic data collection: retrospective review of inappropriate cases (100 for head, chest, abdomen, MSK), every year
Procedure			
	Computer tomography		
	Over sampling	Number of patients	Review of 100 patients for: head, chest, abdomen, MSK every year
	Over phasing	Number of patients	Review of 100 patients for: head, chest, abdomen, MSK every year
	Positioning in the gantry	Number of wrong	Review of 100 patients for: head, chest, abdomen, MSK every year
	CDRLs	% of patients beyond 75% % of patients beyond 50%	If there is a dms: data extraction every 6 months If there is not an automatic data collection: retrospective review: 100 for head, chest, abdomen, MSK, every year
	Repeated examinations	Number of patients with more than 5 CT in a year	If there is a DMS: data extraction every 6 months If there is not an automatic data collection: retrospective review: 100 for head, chest, abdomen, MSK, every year
	CT scan performed without contrast medium when contrast was required	Number of patients	If there is a DMS: data extraction every 6 months If there is not an automatic data collection: retrospective review: 100 for head, chest, abdomen, MSK, every year
	Paediatric	Number of wrong protocols	If there is a DMS: data extraction every 6 months If there is not an automatic data collection: retrospective review: 100 for head, chest, abdomen, MSK, every year
	Pregnant women	Number of misses	If there is a DMS: data extraction every 6 months If there is not an automatic data collection: retrospective review: 100 for head, chest, abdomen, MSK, every year
	Radiography		
		Repeated exposures	Number of repeated exposures Retrospective review: 100 for chest, msk, every year
	Digital radiography data deleted prior to image review	Number of patients	Review of patient examinations with data deleted every year
	Unintended conceptus exposure	Number of misses	If there is a DMS: data extraction every 6 months If there is not an automatic data collection: retrospective review: 100 for head, chest, abdomen, MSK, every year
	Interventional radiology		
	Patient	Number of skin doses managed per year	
	Patient	Threshold for deterministic effects exceeded	Review of patient cases exceeding skin dose threshold every year
	Staff	Number of staff doses managed per year	
Reporting			
	Dose reporting	% of missed	Data extraction from the RIS or from the PACS (check)
General			
		Over exposure	Number of patient dose values managed per year
		Quality control	Number of QC per year (with written reports) and including the pacs and the patient dose management systems

### Use case: dose monitoring

**Fig. 2:**
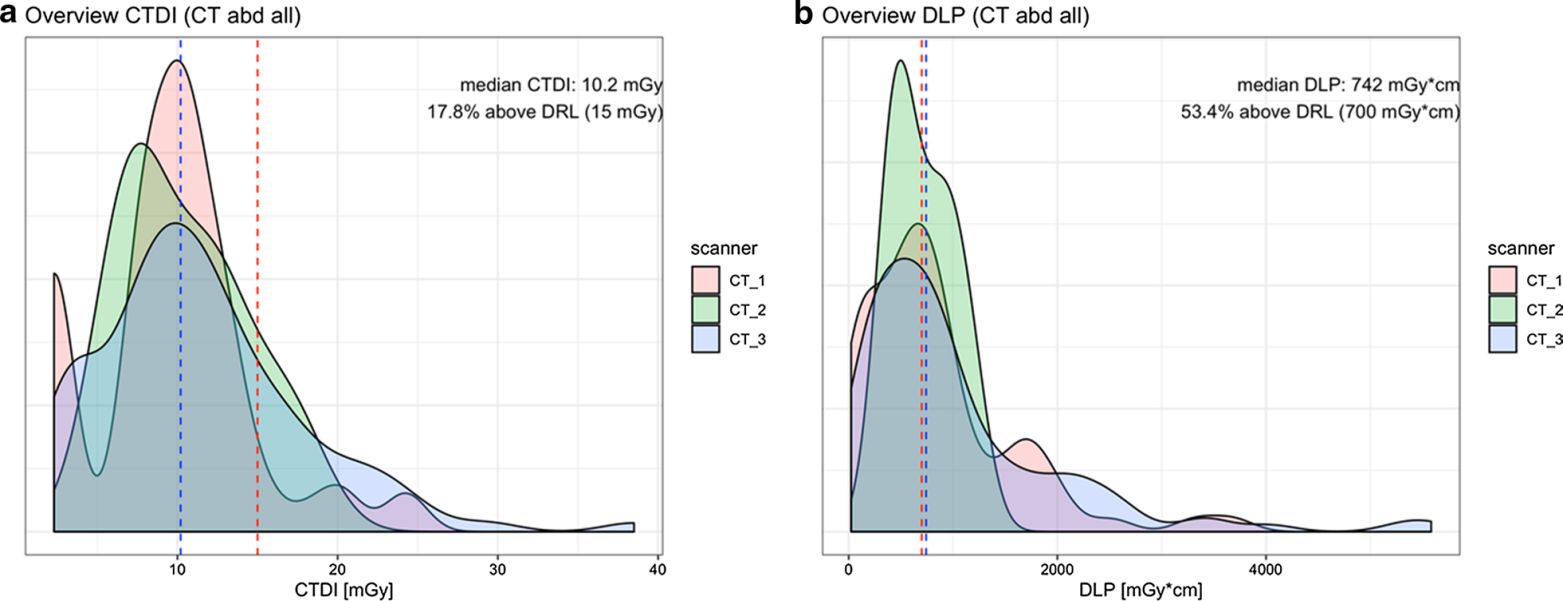
Dose monitoring for a dedicated CT protocol (CT abdomen) with comparison of three different scanners. The red line demonstrates the DRL for regular patients (70 kg). The graph shows that some studies (17,8%) have CTDIs higher than expected, and DLP is often above limits (courtesy: Dr. Daniel Pinto dos Santos, Cologne, DE)

Continuous real-time monitoring of dose levels is relevant, especially in CT, the examples highlight the potential of such a KPI. In this case, there is a mean CTDI of about 10 mGy in one system and about 8 mGy in a second system, and also nearly 18% of the studies have a CTDI above the official DRL (Fig. 2). Based on this information, the responsible radiologist can analyse the reasons, e.g. patients with higher weight.

## Conclusion

A continuous, timely evaluation of several criteria is relevant in the optimisation of radiation protection. KPIs are central components to collect relevant information in regard of patient protection, but also in the protection of staff, e.g. involved with high-dose interventional procedures.

KPIs, which can be recorded automatically and visualised in dashboards, for example, are suitable for this purpose. A selection of indicators covering different areas has been discussed and suggestions made for their implementation.

Due to BSS Directive regulatory requirements, clinical audit is now mandatory in radiology. Clinical audits, however, do not generally lend themselves to continued monitoring (as is needed in some areas of radiation protection). Continuous, timely monitoring of the efficiency of radiation protection can and should therefore be supplemented by further measures.

As shown within this overview, there are different KPIs already available. A more detailed analysis of their applicability and significance would be helpful. A possible extension of criteria and further specification should also be examined. The ESR EuroSafe Imaging Steering Committee will continue to address this issue.

## Abbreviations

CDRL: DRLs based on clinical indication
DMS: Dose management system
KPI: Key performance indicator
